# Somatostatin receptor scintigraphy in sarcoidosis

**DOI:** 10.1186/1479-5876-9-S2-P9

**Published:** 2011-11-23

**Authors:** Lieke SJ Kamphuis, Dik J Kwekkeboom, Jan AM van Laar, Paul LA van Daele, G Seerp Baarsma, P Martin van Hagen

**Affiliations:** 1 Dept. of Immunology, Erasmus University Medical Center, Rotterdam, The Netherlands; 2 Dept. of Internal Medicine, Section Clinical Immunology, Erasmus University Medical Center, Rotterdam, The Netherlands; 3 Dept. of Nuclear Medicine, Erasmus University Medical Center, Rotterdam, The Netherlands; 4 Rotterdam Eye Hospital, Rotterdam, The Netherlands

## Background

Sarcoidosis is a multisystem granulomatous disorder, most frequently involving the lungs, skin or eyes. Somatostatin receptor scintigraphy (SRS) can localize granulomas by binding to somatostatin receptors that are expressed in sarcoidosis. We studied the patterns of uptake on SRS and correlated them to other disease parameters.

## Material and methods

218 patients were studied. The degree of intensity of uptake and localization of sarcoidosis associated lesion (SAL) were determined. The degree of intensity was compared with serum angiotensin converting enzyme (ACE) and serum soluble interleukin-2 receptor (sIL-2R). Elevated levels of serum ACE and sIL-2R have been shown to be associated with active sarcoidosis. Typical patterns on SRS were compared to conventional chest CT-scans and x-rays.

## Results

SRS was negative in 28 patients, 10 patients had one -and 180 patients had more SALs. The degree of intensity correlated significantly with higher serum levels of ACE (p = 0.001) and serum levels of sIL-2R (p <0.0001). An elevated level of serum ACE and sIL-2R shows a sensitivity of respectively 96% and 93% for uptake on the SRS. Mediastinal lesions together with either eye, salivary glands, clavicular or hilar localizations on SRS demonstrated a significant characteristic pattern. All patients with abnormal conventional tests had SRS uptake. Moreover, of 94 patients with normal radiological findings 49 expressed pathological SRS uptake. In 36 of these 49 patients a lung biopsy was taken, which revealed sarcoidosis in 31.

## Conclusions

The degree of intensity in SRS correlates with sarcoidosis activity (ACE and sIL-2R). SRS is more sensitive in diagnosing sarcoidosis, even in patients with normal chest radiology. SRS therefore provides a useful and sensitive imaging technique to monitor organ involvement and therapy in patients with sarcoidosis.

